# Monitoring Disease Severity of Mild Cognitive Impairment from Single-Channel EEG Data Using Regression Analysis

**DOI:** 10.3390/s24041054

**Published:** 2024-02-06

**Authors:** Saleha Khatun, Bashir I. Morshed, Gavin M. Bidelman

**Affiliations:** 1 Department of Electrical and Computer Engineering, University of Memphis, Memphis, TN 38152, USA; 2 Compute Science Department, Texas Tech University, Lubbock, TX 79409, USA; 3 School of Communication Sciences and Disorders, University of Memphis, Memphis, TN 38152, USA

**Keywords:** electroencephalography, event-related potential, mild cognitive impairment, Montreal cognitive assessment, deep learning, ensemble regression

## Abstract

A deviation in the soundness of cognitive health is known as mild cognitive impairment (MCI), and it is important to monitor it early to prevent complicated diseases such as dementia, Alzheimer’s disease (AD), and Parkinson’s disease (PD). Traditionally, MCI severity is monitored with manual scoring using the Montreal Cognitive Assessment (MoCA). In this study, we propose a new MCI severity monitoring algorithm with regression analysis of extracted features of single-channel electro-encephalography (EEG) data by automatically generating severity scores equivalent to MoCA scores. We evaluated both multi-trial and single-trail analysis for the algorithm development. For multi-trial analysis, 590 features were extracted from the prominent event-related potential (ERP) points and corresponding time domain characteristics, and we utilized the lasso regression technique to select the best feature set. The 13 best features were used in the classical regression techniques: multivariate regression (MR), ensemble regression (ER), support vector regression (SVR), and ridge regression (RR). The best results were observed for ER with an RMSE of 1.6 and residual analysis. In single-trial analysis, we extracted a time–frequency plot image from each trial and fed it as an input to the constructed convolutional deep neural network (CNN). This deep CNN model resulted an RMSE of 2.76. To our knowledge, this is the first attempt to generate automated scores for MCI severity equivalent to MoCA from single-channel EEG data with multi-trial and single data.

## 1. Introduction

Memory disorder brings difficulties in performing Basic Activities of Daily Living (BADL) such as walking, eating, dressing, showering, feeding, continence, and entertainment, and Instrumental Activities of Daily Living (IADL) such as preparing meals, solving everyday situations, managing finances, doing laundry, and taking medications [1]. Mild cognitive impairment (MCI) is considered to be the state between the normal cognition and dementia [2]. There are many benefits of early detection of MCI. MCI has a 54% chance of becoming Alzheimer’s disease (AD) or related dementia [3]. MCI reduces the ability of elderly people to perform daily activities and to live an independent life. In some cases, patients suffering from complicated diseases (e.g., Parkinson’s Disease) may have develop MCI later [4], and progression of cognitive impairment monitoring is crucial for their caregivers, doctors, and families. MCI may be associated with cardiovascular disease, metabolic syndrome, type 2 diabetes, sedentary activity, obesity, excess alcohol, and smoking [5].

According to the Alzheimer’s Association, persons who already have cognitive impairment based on clinical observations or have self-reported concerns should undergo a cognitive impairment assessment. For cognitive health screening, they are recommended to undergo any one of the cognitive screening tests: (a) Montreal Cognitive Assessment (MoCA), (b) Mini-Mental State Examination (MMSE), (c) St. Louis University Mental Status Exam (SLUMS), (d) General Practitioner Assessment of Cognition (GPCOG), (e) Mini-Cog, (f) Memory Impairment Screen (MIS), (g) AD8, or (h) Informant Questionnaire on Cognitive Decline in the Elderly (short-IQCODE) [6]. These tests are manual and conducted by experienced clinicians or caregivers. Scientists compared the performances of these cognitive assessment tests and showed that when a patient’s cognitive impairment falls beyond memory impairment, MoCA should be used [7]. Furthermore, some studies suggest that MoCA is more sensitive than the widely used MMSE [7,8]. Researchers have predicted cognitive scores based on physiological data, such as an electroencephalogram (EEG), in a motivation setting, to help monitor a person’s cognitive health easily and at a time when a patient is unable to undergo a manual cognitive assessment test. All of the research related to measuring the severity of cognitive impairment was based on features extracted from a multi-channel EEG system: an EEG power ratio from a 16-channel system by Bennys et al. [9], EEG power spectra and other information from a 16-channel system by Kowalski et al. [10], EEG power of prominent bands from a 20-channel EEG system [11], spectral characteristics of EEG from a 20-channel system [12], an EEG power ratio from a 19-channel system [13], the grand total EEG score from a 16-channel system [14], etc. EEG rhythms are shown to capture brain neural synchronization and functional coupling in Alzheimer’s disease [15]. EEG rhythms can show cortical sources of resting state in Parkinson’s disease related dementia and Alzheimer’s disease [16]. Also, brain oscillatory patterns in MCI due to Alzheimer’s and Parkinson’s disease were detected with high-density EEG [17]. Thus, this work on MCI severity detection by generating MoCA-equivalent scores from a single-channel EEG system in unsupervised settings is novel.

In this work, we have performed objective generation of MCI severity scores based on single-channel EEG data collected from the Fz location. Our analysis is divided into two parts: (a) multi-trial, and (b) single-trial. We followed a multi-trial approach based on classical regression techniques. We used features extracted from grand average event-related potential (ERP) in this case. In the single-trial analysis, we determined MCI severity based on a convolutional deep neural network. We used the time–frequency plot image of each trial as the input for the deep neural network. Our key contribution here is our proposed models based on a multi-trial approach (best for offline system) that uses multiple data in aggregate, and a single-trial approach (best for real-time system with streaming data) that uses each new datum in isolation to predict the severity of cognitive impairment to continually assess cognitive health.

## 2. Method

### 2.1. Participants

Twenty-three older adults were recruited in a study which was designed to observe the aging effect on the auditory system and to evaluate the cognitive performance of the subjects. EEG data were collected in this study from an experiment where twenty-three older adults (age ranged from 52 to 86 years; mean ± standard deviation: 70.2 ± 7.2 years) received auditory stimuli. All the participants had no known history of neurological or psychiatric illness, and they all were strongly right handed [18]. We anticipate that there would be no change in outcome with left-handed patients; however, there were no subjects available at the time of recruitment, which can be a focus of a future study. During the participant recruitment process, exclusion criteria were followed based on age, hearing loss, musical training, and handedness [19]. Written consent under the protocol approved by the Baycrest Centre Ethics Committee (IRB number: REB #06-31) was collected from each participant before data collection, and participants were compensated for their time after data collection. The cognitive health status of the participants was assessed by the well-established cognitive screening tool called the Montreal cognitive assessment (MoCA) test [20]. In this cognitive screening test, among all the participants, fifteen older participants (8 male, 7 female) were found to have normal cognition (MoCA score ≥ 26 points; mean ± standard deviation: 27.6 ± 1.18; range: 26–30), and eight participants (4 male, 4 female) were assessed to have MCI (MoCA score < 26; mean ± standard deviation: 23.0 ± 1.85; range: 20–25). Also, it is important to mention that in general, patients with Alzheimer’s disease or more severe dementia generate MoCA scores in the range of 11.4 to 21 [20].

### 2.2. Study Design

This study was designed so that the subjects would hear five auditory stimuli. Stimuli were constructed with a perceptual phonetic continuum from∕u/to∕a/by, varying the first formant (F1) frequency parametrically between 430 and 730 Hz over five equal steps (for further stimulus details, see [21]). The synthetic five-step vowel continuum (denoted hereafter as “vw1-5”) was built in a way such that each token of 100 ms sound would differ minimally acoustically during hearing, and would still be perceived categorically [22,23]. As we are aware that hearing loss due to aging may alter auditory-evoked potentials from the cortical region [23], and it may affect the response of the participant, audiometric testing was performed. However, the audiometric testing demonstrated that hearing thresholds were not distinguishable between the groups (Normal and MCI) at octave frequencies between 250 and 4000 Hz [19], which is well outside of the bandwidth of the stimuli.

### 2.3. Collecting Data and Processing Event-Related Potentials

The data collection technique and response evaluation employed in this study are similar to the methodologies reported in previous research [24]. During EEG recording, the participants underwent 200 trials for each sound token within an electro-acoustically shielded chamber (Industrial Acoustics, Inc., North Aurora, IL, USA). Stimuli were delivered through earphones (ER-3A, Etymotic Research, Elk Grove Village, IL, USA) in both ears at an intensity of 83 dB SPL. To mitigate electromagnetic stimulus artifacts, extended acoustic tubing (50 cm) was utilized [25]. Sound tokens were presented randomly, and participants were tasked with rapidly categorizing them with a binary response (“u” or “a”). Each sound token had a duration of 100 ms, with 10 ms of rise and fall time to minimize spectral splatter [23]. Following the participants’ response, an inter-stimulus interval (ISI) followed randomly between 400 and 600 ms (20-ms steps, rectangular distribution) to prevent subjects from anticipating subsequent stimuli [26]. EEG data were captured from the subjects using SynAmps RT EEG amplifiers (Compumedics Neuroscan, Charlotte, NC, USA). This system consists of 64-channel EEG data collection electrodes. The participants were in a magnetically shielded room while the data collection occurred. EEG data were recorded between an electrode placed high on the forehead at the hairline with reference to linked mastoids. For recording auditory-evoked potentials of cortical origin, the Fpz—A1/A2 montage was considered optimal [27,28]. Throughout the data collection procedure, contact impedances were maintained below 3 kΩ, the EEG signal was sampled at 20 kHz, and raw signals were filtered with a band-pass filter with a passband range of 0.05 Hz to 3500 Hz.

**Figure 1:**
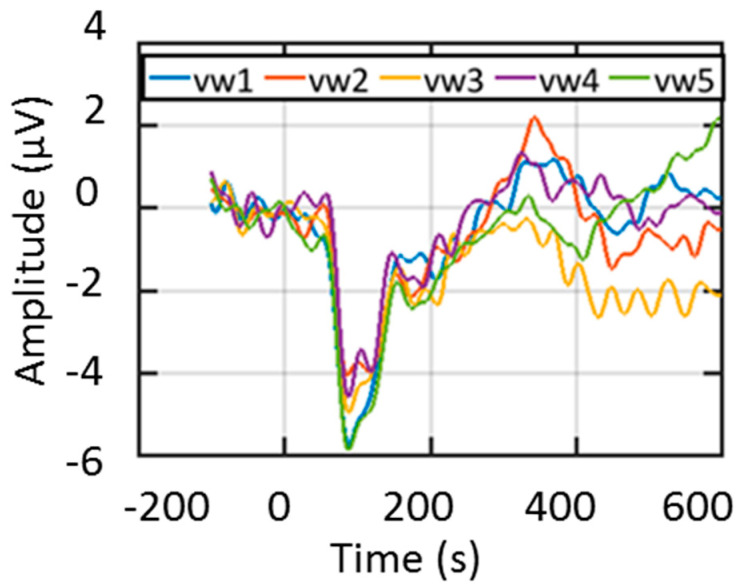
A representative multi-trial ERP from a subject with auditory stimuli of vw1, vw2, vw3, vw4, and vw5.

The EEG data then underwent processing using ERPLAB, an open-source toolbox operating within the MATLAB environment [29]. Each EEG epoch consisted of a 700 ms (100 ms pre-stimulus and 600 ms post-stimulus) interval, as depicted in Figure 1 [3]. The pre-stimulus region was used as the reference, and the subtraction method was used for baseline correction [30]. Trials surpassing ±50 μV were excluded from analysis by the threshold method, as they were likely tainted by various artifacts, such as eye blinks and eye movements, as mentioned in previous studies [31,32]. Artifact-free epochs from each auditory stimulus were utilized to compute the grand average, called event-related potentials (ERPs), representing the average across 200 trials. Subsequently, the grand average ERP underwent bandpass filtering within the range of 0 to 30 Hz, aligning with a priori knowledge of ERP bandwidths and the stimuli [33]. A visual representation of the extracted ERP from multiple trials of a representative subject (ID 3610) is illustrated in Figure 1.

## 3. Multi-Trial Analysis

### 3.1. Extraction and Ranking of Features

**Figure 2:**
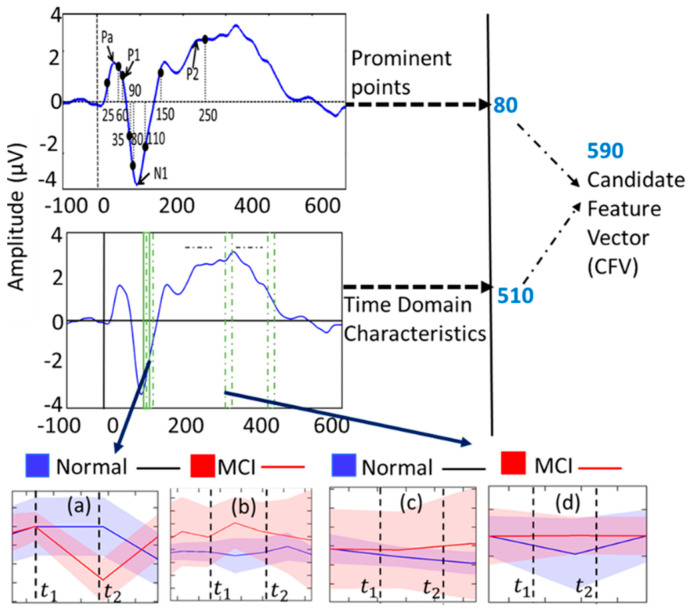
Schematic of the feature extraction process: (**a**,**c**) are slopes and (**b**,**d**) are covariances for two different time windows from two timeframes as shown.

Multi-trial analysis uses multiple trial data to predict the MoCA score offline, while single-trial analysis uses single trial data to predict MoCA in real time. We extracted a total of 590 candidate features from the prominent points of the ERP and the characteristics in both the time and spectral domains. From this pool, the top 25 features were selected for integration into the classification models, determined by ranking using the random forest algorithm. The cortical auditory-evoked responses indicated that prominent ERP points possess discriminatory power in distinguishing between normal and MCI stages among older adults [34]. Hence, we included ERP prominent points, such as Pa, P1, N1, and P2, in the candidate feature vector (CFV). These ERP prominent points were defined as peaks within the intervals {25–35 ms}, {60–80 ms}, {90–110 ms}, and {150–250 ms}, respectively [3], as illustrated in Figure 2. The CFV incorporates not only the peak amplitudes and their corresponding latencies but also the mean amplitudes of the intervals containing these prominent points. This inclusion is based on their recognized significance in distinguishing between groups in experiments involving evoked responses [35]. Furthermore, the CFV encompasses the relative powers in EEG bands (i.e., delta, theta, alpha, and beta rhythms), known to be valuable in classifying normal, mild cognitive impaired, and Alzheimer’s disease groups [36]. This addition results in a total of 16 features calculated from the ERP prominent points for each stimulus, contributing to a cumulative 80 feature points in the CFV.

Given the rapid temporal changes in ERPs during the application of a stimulus, it is crucial to capture the high-resolution variations in time-domain characteristics. To achieve this, we implemented a 25 ms window with a 50% overlap across the entire ERP signal, as illustrated in Figure 2. The sliding window methodology enables the observation of time-domain property variations, a factor previously shown to be significant in distinguishing between MCI and normal subjects in earlier studies [19]. A total of 107 time-domain characteristics, including signal statistics, correlation properties, and entropies, were computed from each window using the open-source software package “HCTSAtool” [29] in MATLAB (r2018b). For details on the extracted time-domain characteristics, please refer to [31,32,33]. To assess the variation in time-domain characteristics over time, we calculated the slope and the coefficient of variation (CV) from two feature points at each time stamp for every stimulus. In the selection process for the CFV, feature points demonstrating both intra-class similarity and inter-class variability, determined through visual inspection, were included. For instance, Figure 2a,b displays the slope and CV of a time-domain characteristic (computed by HCTSAtool as “proportion of data within two-standard deviation of mean” [34,35,36]) for the normal and MCI groups, revealing intra-class similarity within the shaded regions but inter-class variability, thereby making it a CFV feature point. Similarly, the slope and CV in Figure 2c,d at different time frames exhibit both intra-class similarity and inter-class variability, meeting the criteria for inclusion in the CFV. Out of all time-domain feature points, 510 fulfilled the conditions mentioned above, securing their place in the CFV.

The CFV, comprising a total of 590 features (80 from prominent points and 510 from time-domain characteristics), underwent ranking by the lasso regression algorithm. From this comprehensive set, the top 13 features were selected for integration into the regression methods. The implementation of the lasso regression algorithm was carried out using MATLAB. Lasso serves as a regularization technique designed to identify optimal features in a regression problem, thereby reducing the number of features within a large feature set [37]. For a given value of λ, lasso solves the below mathematical problem:

$$\underset{\beta_{0},\beta }{\min}\left(\frac{1}{2N}\sum_{i=1}^{N}{\left(y_{i}-\beta_{0}-x_{i}^{T}\beta \right)}^{2}+\lambda \sum_{j=1}^{p}\left|\beta_{j}\right|\right)$$

Here, *N* is the number of observations, *y_i_* is the response at observation *I*, *x_i_* is data, a vector of *p* values at observation *i*, *λ* is a positive regularization parameter corresponding to one value of Lambda, $\beta_{0}$, β are scalar parameters, and *p* is vector parameters.

**Figure 3:**
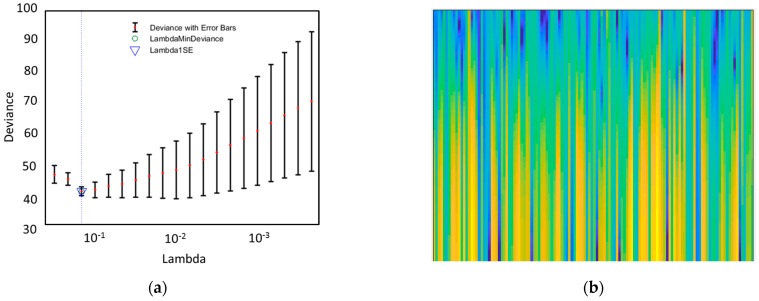
(**a**) Lasso regression-based feature selection: five-fold cross-validated mean squared error/deviance vs. lambda. (**b**) time–frequency image feature sample.

According to Figure 3a, when the lambda is close to 10^−1^, the cross-validated deviance is the lowest and the number of features that have generated the deviance is 13.

### 3.2. Regression

As the MoCA scores are bounded 0–30, instead of straight linear regression, multivariate regression with logit link function, ensemble regression, support vector regression, and ridge regression were analyzed. We performed five-fold cross-validation to ensure the robustness of the model and the reliability of the results. 

## 4. Single-Trial Analysis

### 4.1. Feature Extraction 

For the single trial analysis, we considered each trial length from the onset of the stimulus to the response of the subjects. We excluded those responses which were less than 0.2 ms or greater than 1.5 ms [19], or where the range of the magnitude of the signal was greater than 100 µV, as they might have been contaminated by artifacts [38]. We then performed a time–frequency (TF) analysis of each individual trial from 0 to 100 Hz frequencies. We collected the generated images of the TF plots in a png format and used them as inputs for deep neural network analysis. The function pspectrum.m of MATLAB (2018a) was used to extract the TF features.

### 4.2. Deep Neural Regression and Bayesian Optimization

**Figure 4:**
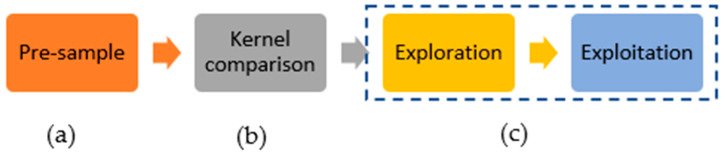
Deep learning classification steps: (**a**) Pre-sample processing, (**b**) Kernel comparison (see Table 1), and (**c**) Exploration and Exploitation stage.

We had a total of 2848 samples as inputs to the deep learning network. We constructed the deep neural network with the help of the keras package from Python. We used a sequential model, where we constructed three sets of a convolutional 2D layer and max-pooling layer, with a ‘relu’ activation layer. Then, we used the flatten layer, two dense layers with a ‘tanh’ activation layer in between. To find out the optimal convolutional filter size, dropout rate, and number of epochs, we used Gaussian process-based Bayesian optimization. The whole process of the Gaussian process-based Bayesian optimization can be divided into four steps (Figure 4). For convenience, in our explanation, we will explain the third and fourth steps together. Thus, the three steps are: (a) pre-sample, (b) kernel comparison, and (c) exploration and exploitation. In the pre-sample stage, we defined a uniform distribution for each of our hyperparameters (convolutional filter size, dropout rate, and no. of epochs). Each time, we randomly sampled from the uniform distributions without repetition and used them in the deep neural network to obtain the cross-validated root mean square error. We repeated the process five times in the pre-sample stage. Then, we entered into the kernel comparison stage. We used five kernels: (i) Matern, (ii) Radial Basis Function, (iii) Rational Quadratic, (iv) Exponential Sinsquared, and (v) Dot Product to compare the performance of the sample fitting into the Gaussian process. The kernel performances in terms of the sum square error (SSE) are given in Table 1.

**Figure 5:**
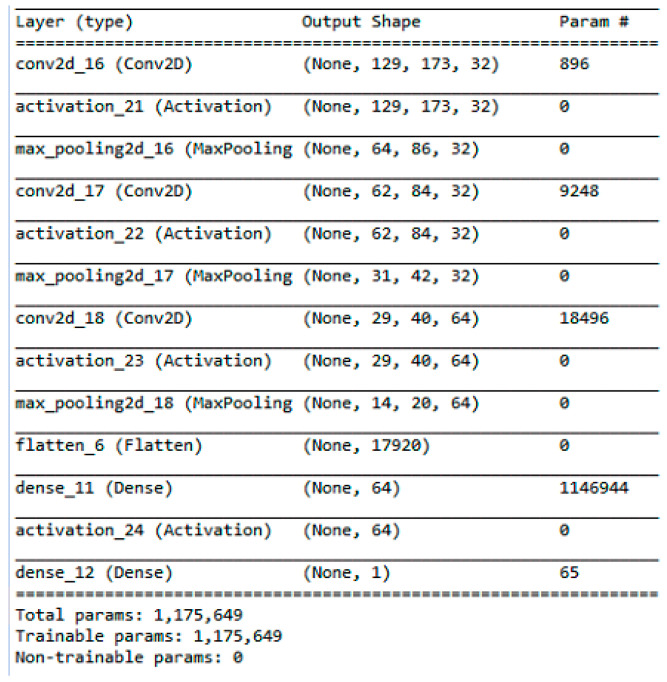
Convolutional deep neural network for regression: layers and their parameters.

We used the Rational Quadratic kernel for the rest of the Bayesian optimization process. In the exploration stage, we defined the Gaussian process with the *y* kernel. We fitted it with pre-sampled data. Then, we used the following acquisition function to calculate our next sample.

$$EI\left(x\right)=\left\{\begin{array}{c} \left(\mu \left(x\right)-f\left(x^{+}\right)\right)\Phi \left(Z\right)+\sigma \left(x\right)\phi \left(Z\right)\;\;\;\;\;\;\;\;if\;\sigma \left(x\right)>0\;\\ \;\;\;0\;\;\;\;\;\;\;\;\;\;\;\;\;\;\;\;\;\;\;\;\;\;\;\;\;\;\;\;\;\;\;\;\;\;\;\;\;\;\;\;\;\;\;\;\;\;\;\;\;\;\;\;\;\;\;\;\;\;\;\;\;\;\;\;\;\;if\;\;\sigma \left(x\right)=0\;\; \end{array}\right.$$

$$Z=\frac{\mu \left(x\right)-f(x^{+})}{\sigma (x)}$$

where *x* is the array of possible samples that can be taken in the next steps, $\mu \left(x\right)$ and $\sigma \left(x\right)$ are the mean and standard deviation of the output values returned by the Gaussian process against *x*, $f\left(x^{+}\right)$ is the lowest RMSE obtained so far, and $\Phi \left(Z\right)$ and $\phi \left(Z\right)$ are the PDF and CDF of the standard normal distribution, respectively [39]. We took the sample with the highest EI value according to equation *Z* for deep neural network implementation. We repeated the process fifteen times and concluded the exploration stage. As we obtained many similarly performing samples in the exploration stage, we did not want to use some samples. Finally, we selected the convolutional filter size, dropout rate, and epoch. The detail parameters of the model are depicted in Figure 5. In the Bayesian optimization phase and in the final phase, after constructing the deep neural network model, we compiled it with an ‘adadelta’ optimizer. We evaluated the model with five-fold cross-validation.

## 5. Results

**Figure 6:**
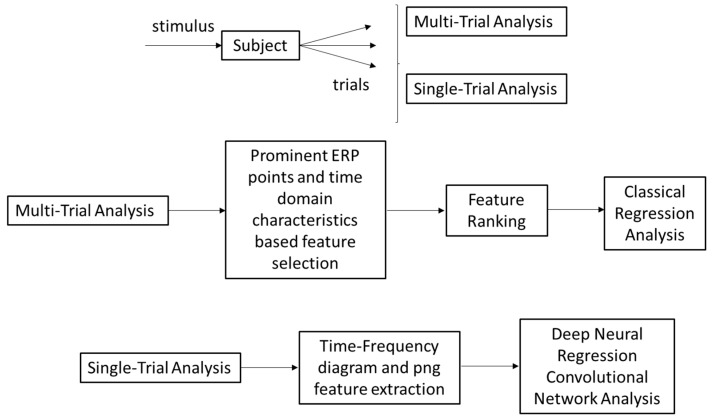
Flow Diagram of the method for single- and multi-trial analysis for individual or aggregate MoCA score prediction.

In the multi-trial analysis, we used the top 13 features, which were decided by the outcome of the lasso regression-based feature extraction techniques in all the regression techniques mentioned in the regression section, to predict the MoCA scores of the neural measures (Figure 6). To compare the regression techniques used in the multi-trial analysis, we used the root mean square error (RMSE). The scores are reported in Table 2.

The regression methods MR, ER, SVR, and RR generate five-fold cross-validated RMSE values of 30.9, 1.6, 0.27, and 2.61, respectively. Based on the RMSE, support vector regression (SVR) performs the best out of all of the techniques in our study. The second-best technique is ensemble regression (ER) in this work. We have performed residual analysis to check the robustness of these models.

**Figure 7:**
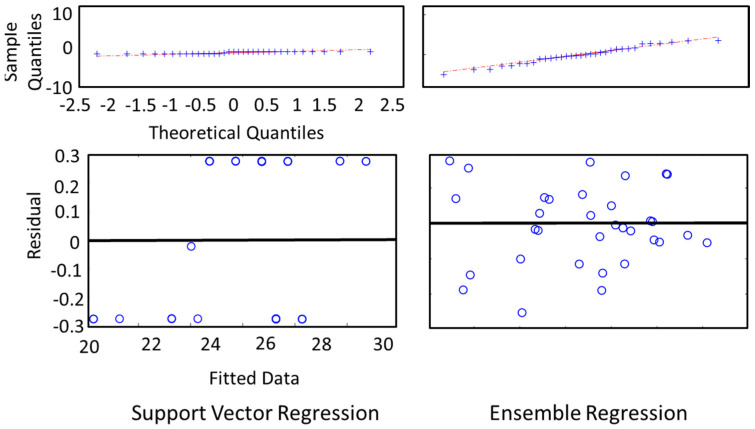
Residual analysis for support vector regression (SVR) and ensemble regression (ER) used in multi-trial analysis.

In Figure 7, we depict the residual analysis results of the two best-performing regression models (SVR, ER). For each regression model, we presented two graphical analysis: (a) sample quantiles vs theoretical quantiles of the residuals or quantile–quantile plot (qqplot), and (b) residuals vs fitted data by the regression model. The qqplot of the residuals is generally used to verify the assumption that the residuals are normally distributed. As both of the models show normal patterns (an approximately straight line) in the qqplot, the models pass the normality test. We further analyzed the residual vs fitted plot. A good regression model should be homoscedastic, which means its residual is uncorrelated, uniform, and random over the fitted or predicted value. The obtained results fit a good regression model criterion. 

The plot of SVR shows a pattern, whereas the plot for ER shows randomness of the residual. After this analysis, we found that the ER model for these data is superior to SVR, although SVR gave a lower RMSE than ER (Figure 7). This is because the ER model shows robustness in all the analyses in all the models we experimented with. For the implementation of ER and RR, we used MATLAB, and for implementing MR and SVR, we used several packages such as “e1071”, “caret”, etc. For residual analysis, we also used MATLAB.

**Figure 8:**
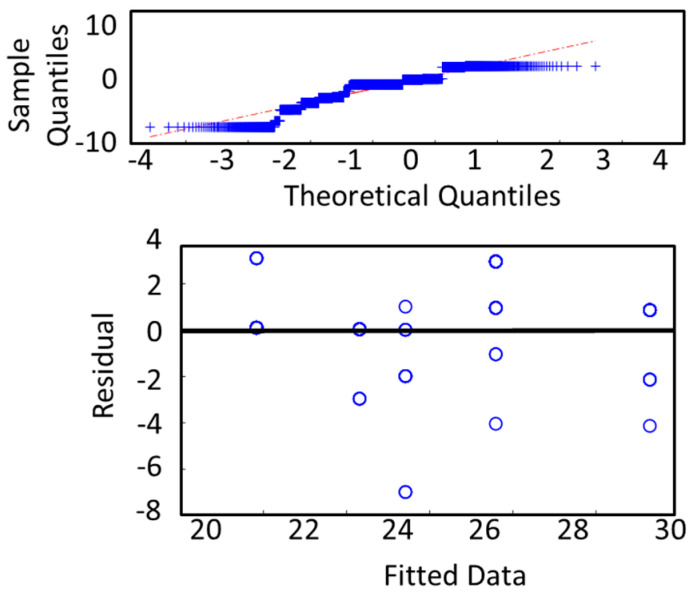
Residual analysis for deep neural network regression model used in single-trial analysis.

In the single-trial analysis, we calculated the cross-validated RMSE and mean absolute error (MAE) for the constructed convolutional deep neural network. To check the robustness of the model, we also carried out a residual analysis which is presented in Figure 8. We tried a different combination of convolutional, max-pooling, and activation layers and calculated the RMSE and MAE for all of them. The best result is reported here (Table 3). Our deep neural network regression model was able to achieve an RMSE of 2.76 and an MAE of 1.81. 

Finally, in Figure 8, we show the residual analysis of the deep neural network. We observed that the qqplot (sample quantiles vs. theoretical quantiles) of the residuals can be approximated as linear with a small deviation. So, the model maintains the normality or monotonicity assumption. Also, the residuals vs the fitted plot shows that the residuals do not follow any pattern with the change in the fitted value. Thus, these might be uncorrelated. 

In comparison, the results demonstrate that multi-trial analysis can be utilized for MoCA score prediction with sufficient confidence. In particular, SVR has a very small MASE (0.27). Furthermore, single-trial analysis can also be utilized to predict the MoCA score with a deep neural network. However, the RMSE is higher (2.76). Nonetheless, single-trial analysis might be more suitable in practical cases, where patient profiles can be updated during each visit, using a single-trial approach to predict MoCA objectively. This can be further explored for in-home MoCA score prediction applications for more granular data points.

## 6. Conclusions

Although MCI detection has been investigated by researchers in recent years at great length, MCI severity measurement has not been studied intensely; although, the ability to measure such a metric objectively and automatically could improve MCI early diagnosis, prognosis, and patient management. In the current study, we generated the MoCA score from the single-channel EEG data using unsupervised algorithms, which can help to assess cognitive impairment automatically and continually with a low-cost, portable, and less burdensome EEG system. We carried out multi- and single-trial analysis, and the results of both cases were promising. Multi-trial analysis uses multiple trial data, while single-trial analysis uses single trial data, to predict the MoCA score. In the multi-trial analysis, we applied several classical regression models and found that the ER (ensemble regression) performed best with an RMSE of 1.6. In the single-trial analysis, our constructed convolutional deep neural network generated an MoCA score with an RMSE of 2.76. To the best of our knowledge, we are the first to generate automated scores equivalent to (manual) MoCA scores from single-channel EEG data. We have performed this with both multi-trial and single-trial data from a single-channel EEG. We believe that our current study has a broad scope in portable cognitive health study, precision medicine, mobile health (mHealth), and smart and connected community (SCC) research. For instance, access to objective and more granular MoCA scores can be beneficial to improve diagnosis, prognosis, and efficacy monitoring of therapy of Alzheimer’s disease. This can allow at-home monitoring systems or remote monitoring systems for MoCA scores beyond traditional clinical settings. However, the implications of the availability of more granular MoCA scores should be examined with consideration of invasion of privacy, disparity, and ethical implications.

## Figures and Tables

**Table 1:** Kernel performance of data fitting in gaussian process.

Kernel	SSE
Matern	5.45 e−19
Radial Basis Function	5.45 e−19
Rational Quadratic	3.59 e−19
Exponential Sine-squared	2.48 e0
Dot Product	54.14 e0

**Table 2:** RMSE for regression methods used for multi-trial analysis.

Method	RMSE
Multivariate Regression (link function = logit) (MR)	30.9
Ensemble Regression (ER)	1.6
Support Vector Regression (SVR)	0.27
Ridge Regression (RR)	2.61

**Table 3:** RMSE and MAE for deep neural network used in single-trial analysis.

RMSE	MAE
2.76	1.81

## Data Availability

Data are contained within the article.
